# The Enamel Organ

**Published:** 1897-05

**Authors:** 


ARTICLE II.

### THE ENAMEL ORGAN.

The first recognizable stage in the process by which teeth are envolved in the higher animals is, I need scarcely remind any one here, the growth of a band of cells derived from the surface epithelium, which penetrates the embryonic connective tissues of the fœtal jaw to a considerable depth, and is now known as the tooth band. From this primitive tooth band an epithelial bud, called the enamel organ, is produced at the site of each future milk tooth. The enamel organ is the formative organ. It calls the tooth into existence, and probably determines its shape and size, even although, as in several animals, it forms no part of the tooth finally produced.

A tooth band, theoretically, is constructed to originate a constant succession of teeth by the continuous budding of enamel organs from its lower margin. This happens in many of the lower vertebrata, but in mammalia the band becomes exhausted after the production of a second set of germs. In rare instances a third successional germ buds from a part of the tooth band which has already originated two others. Such a germ may remain abortive. or may continue to develop, and become either a supernumerary tooth or an odontome. The former conditions, the budding of a third enamel organ which undergoes no further development, is perhaps not so very uncommon, and is a point which should be settled as regards the human subject by examining a series of embryos during the later months of intra-uterine life. I have some sections at that period which certainly seem to show this third budding from the tooth band, but not sufficient to indicate its frequency.

The enamel organ as first produced is a solid bud of epithelial cells, but the latter, as the bud grows, are rapidly differentiated into the four characteristic layers of the enamel organ. The outermost or peripheral layer of cells are elongated. They are continuous with, and correspond to the important cubical layer of cells constantly found at the base of a stratified epithelium, and they fully retain their character of importance in the enamel organ. Over the deep half of the bud these cells constitute the internal enamel epithelium, from which the enamel fibres are developed; whilst those which cover the superficial half become the external enamel epithelium, the chief function of which seems to be the separation from the blood of those constituents of enamel which are elaborated by the internal epithelium. I think we should keep in mind this early association between the two layers of the enamel organ and their common derivation from the active cubical layer of the surface epithelium, because the value of the external layer is likely to be lost sight of, though, as I have frequently noticed in the germs of the sheep, ox and other animals, the character of its growth and the high vascularity of the papillary connective tissue associated with it suggest that it has useful work to do. The other two layers of the enamel organ are the enamel jelly, and the stratum intermedium. I believe there is a general agreement that the stellate cells are not functional, and that they merely fill a space which may be readily occupied by the growing enamel. As regards the cells of the stratum intermedium, the generally, but not universally accepted view that they recruit the internal epithelium as the surface to be covered grows larger, seems to me to be correct.

To return to the internal epithelium itself, we are still in want of exact information as to the process by which it is changed into enamel. The question as to whether the fibers are the result of secretion or conversion is not yet definitely settled; but to my mind it is of much less importance to decide this point than it is to recognize that enamel is certainly the outcome of some change in the cells themselves, and not a change which is effected by the cells upon an intercellular matrix. Herein lies the essential distinction between enamel and dentine. Dentine, like all connective tissues, has a matrix; enamel, like all epithelial tissues, has none. No doubt between the cells there is a certain amount of intercellular substance, which serves to cement them together, but this is in no way comparable to a connective tissue ground substance. In the end this cement becomes calcified, but only imperfectly, as is shown by the readiness with which acids act upon it and cause the fibers to fall apart. The recognition of this important fact in the construction of enamel opens the way to understand some of its peculiarities. One of these is the occurrence of the well-known cavities on the dentinal aspect of the enamel with which dentinal fibrils frequently communicate. The cavities have been variously stated to be within or between the fibres. The former is an irrational theory, and is not likely to find much acceptance; indeed, all tubes or spaces occuring in an epithelial structure must almost of necessity be placed between and not within the cells. We can best understand these enamel tubules, as they are met with in mammalin teeth, by examining microscopically germs in an early stage of development and after very careful fixation. On the first appearance of the layer of odontoblasts these cells are seen to be separated from the ameloblasts by a narrow band of transparent tissue, due to a change in the outer border of the pulp matrix. The transparent condition of the pulp matrix is evidently owing to the action of the odontoblasts, and is, indeed, merely the first stage in the formation of dentine matrix into which it will shortly be fully transformed. This first band of dentine matrix lying between the odontoblasts and the ameloblasts is not so sharply defined and cut off from adjacent tissues as superficial examination might lead one to image. Mummery has shown—and I fully endorse his views—that it sends processes between the odontoblasts which communicate with the fibrillar matrix of the pulp.

The presence of these processes is unquestionable, as they may be readily seen in certain stages of development in properly prepared sections. Now not only are there the processes described by Mummery, but the dentine matrix is not even sharply cut off from the line of ameloblasts. You may see in some sections that it sends arms of processes up between the enamel cells as it does down between the odontoblasts, keeping them apart, and leaving between them when they calcify elongated spaces filled with dentine matrix. That processes of dentine matrix thrust up between the enamel prisms should never calcify is certainly nothing surprising when one remembers that the first layer of dentine usually only calcifies imperfectly, being characteristically the site of the interglobulor spaces of Tomes.

If we accept this as a reasonable explanation of those spaces in enamel which are met with in close contact with the dentine, and admit the general principle that all spaces or tubes in enamel are between and not within the prisms, then the structure of genuine tubular enamel seems less difficult to understand. It is clear that any imperfect approximation of enamel cells must leave space between the prisms which can only be filled with an indefinite intercullular substance, or possibly by further prolongations of dentine matrix, and in neither case is it likely that such interprismatic matter would become calcified, because on the one hand it is too far removed from the influence of the odontoblasts, and on the other because the calcifying energy of the ameloblasts is almost entirely expended upon their own internal petrifaction. I would therefore suggest that tubular enamel is an enamel in which there is an excessive amount of intercellular substance only imperfectly calcified, and much as it looks like tubular dentine, it is really formed on an exactly opposite plan. The one is a negative and the other a positive picture. In dentine the cells occupy the tubes, and the intercellular substance becomes the solid calcified matter; in enamel the tubes are represented by the intercullular substances becomes the solid calcified matter; in enamel the tubes are represented by the intercellular substance, whilst the cells become the solid calcified matter.

Tubular enamel is regarded as one of the rarer dental tissues, but—at any rate in fish—I am under the impression that it is sufficiently common, and is rather the rule than the exception. Take, for instance, the whole series of the elasmobranch fishes. In the development of their teeth an enamel organ plays a very prominent part. The whole surface of the tooth germ is covered with long and evidently functional ameloblasts, and when it is necessary to decalcify there is a space between the ameloblasts and the dentine matrix which could hardly have been occupied by anything but formed enamel. A study of the development of such teeth would lead one to assume that enamel would be well represented in the fully formed structure, yet they are described as follows by Tomes :—"A central body of osteo-dentine, the outer portion of which has dentinal tubes so fine, regular, and closely packed as to merit the name of a hard unvascular dentine, and over this again a thin varnish of enamel. (?)" Let me call your attention to a few sections of shark and ray teeth. In all you will notice that the main body of dentine is covered over with a further layer, which is distinctly differentiated from that beneath it by a line of demarcation. This latter, though partly obliterated in older teeth, becomes, in some immature specimens, a line of separation, allowing the cap of so-called hard dentine to come away from the rest. The cap is thickest at the apex of the tooth, and shades away at the base. It has, in fact, the shape and general appearance of a cap of enamel. Moreover, the external "thin varnish of enamel" is part and parcel of it, there being not the slightest indication of any lineof demarcation such as should be more or less visible between enamel and dentine. Hence I am inclined to regard the whole of this outer layer of calcified tissue as enamel, but of the tubular variety; and if tubular enamel is constant in the elasmobranch fishes it is tolerably certain to be not uncommon in other classes.

A further point of interest in connection with the enamel organ is the question of its vascularity. This point has been raised by Professors Howes and Poulson, and is one which ought to admit of definite settlement. I am glad to know that Mr. Woods is preparing to investigate the subject. Up to the present I have never yet seen a vessel in the enamel organ, though I frequently examine this structure in various animals, and therefore hold to the commonly accepted view, that it, like other epithelial structures, is non-vascular. Concerning this question I have rather occupied myself in trying to ascertain how two such observers, if they are mistaken, could have come to hold such an opinion. It seems to me that a mistake might rather easily arise, for there is often a stellate-celled connective tissue just outside the enamel organ, which is almost indistinguishable from the enamel jelly, except for its vascularity. Over the apex of a tooth the internal and external epithelium soon come together, so that one frequently sees a tooth germ in which the apex is embedded in a stellate-celled connective tissue; while the sides are surrounded with enamel jelly. Under such circumstances the two tissues might readily be assumed to be two parts of the same structure, and the vessels of the former could hardly pass without observation. The similarity is rendered more complete by the line of condensed tissue of the sac appearing in section not unlike an external enamel epithelium undergoing atrophy. An examination of some of my sections will show that in small animals a careful discrimination must be exercised to distinguish between the two structures, though in the wide expanse of enamel jelly visible in the large germs of sheep and oxen no such mistake can be made, and it may be positively asserted that in these animals the enamel organ is non-vaccular.

Finally, in connection with the enamel organ, having made further observations in regard to Nasmyth's membrane, I should like to add a few remarks to what I said on a former occasion. Originally I experienced some difficulty in isolating and staining the structure. This is now readily accomplished in the following manner: A fresh unworn tooth is taken immediately after extraction and placed in a phloroglucin decalcifying solution for a few minutes. As soon as the membrane begins to separate the tooth is removed and well washed in several changes of water. It is then stained in Ehrlich's acid hæmatoxylin, again washed, and placed in an aqueous solution of eosine. Finally the membrane is stripped from the tooth and mounted in Farrant. By this process a permanent preparation of Nasmyth's membrane may be made within a quarter of an hour after the extraction of a suitable tooth and one in which the nuclei of the epithelial cells are often brilliantly stained. Under the microscope the membrane is seen to consist of two layers, the outermost being composed of large flattened epithelial cells beneath which is a thin translucent pellicle usually marked with hexagonal impressions derived from the ends of the enamel prisms. Hence I think there can be no doubt that Nasmyth's membrane is a remnant of the enamel organ, and not, as advocated by Tomes, a thin layer of cementum. On a former occasion I gave my reasons for not accepting the observations offered by Tomes in support of his view as proof that that view was correct. It is not necessary to repeat them; the specimens may be left to speak for themselves.—PAUL, *Dental Record.*

